# Association Between Albumin‐to‐Globulin Ratio and Hashimoto’s Thyroiditis: A Cross‐Sectional Study Based on NHANES 2007–2012

**DOI:** 10.1155/ije/6645036

**Published:** 2026-05-13

**Authors:** Wenjun Wu, Jing Jin, Nana Qin, Dutian Xu, Fangxu Liu, Baoyin Li, Yuanchen He

**Affiliations:** ^1^ Department of Cardiovascular Surgery, General Hospital of Northern Theater Command, Shenyang, Liaoning, China, syjqzyy.com; ^2^ College of Traditional Chinese Medicine, Changchun University of Chinese Medicine, Changchun, Jilin, China, ccucm.edu.cn; ^3^ Department of Neurology, Hangzhou Fuyang Hospital of Traditional Chinese Medicine, Hangzhou, Zhejiang, China; ^4^ Graduate School, Liaoning University of Traditional Chinese Medicine, Shenyang, Liaoning, China, lnutcm.edu.cn

**Keywords:** albumin-to-globulin ratio (AGR), autoimmune activity, Hashimoto’s thyroiditis (HT), inflammation biomarker, TgAb, thyroid autoantibodies, TPOAb

## Abstract

**Background:**

The albumin‐to‐globulin ratio (AGR) is an inexpensive and routinely available laboratory index that may reflect nutritional status and systemic immune activity. However, its association with Hashimoto’s thyroiditis (HT) and thyroid autoantibodies has not been well characterized in population‐based samples. We investigated the associations of AGR with HT and thyroid autoantibody levels in US adults.

**Methods:**

We analyzed 10,423 participants from the 2007–2012 National Health and Nutrition Examination Survey (NHANES). Multivariable logistic regression was used to evaluate the association between AGR and HT. Smooth curve fitting was applied to assess the shape of the associations. We examined relationships between AGR and thyroid autoantibodies and performed sensitivity and subgroup analyses to assess robustness.

**Results:**

In fully adjusted models, each one‐unit increase in AGR was associated with a lower likelihood of HT (OR = 0.70; 95% CI: 0.54–0.91; *p* < 0.05). Curve‐fitting analyses supported a linear inverse association between AGR and HT. AGR was also inversely associated with thyroid peroxidase antibody (TPOAb) levels (*β* = −11.33; 95% CI: −17.91 to −4.75; *p* = 0.0007), whereas the association with thyroglobulin antibody (TgAb) was not statistically significant (*β* = −4.23; 95% CI: −10.88 to 2.43; *p* = 0.2129). Sensitivity and subgroup analyses yielded consistent results.

**Conclusion:**

Lower AGR was associated with higher odds of HT and higher TPOAb levels in a nationally representative US sample. As a routinely available composite index, AGR may be useful for HT risk assessment and early identification at the population level. These findings are observational and hypothesis‐generating; prospective and mechanistic studies are warranted to confirm temporality and clarify underlying pathways.

## 1. Introduction

Hashimoto’s thyroiditis (HT), also referred to as chronic lymphocytic thyroiditis, is among the most prevalent autoimmune thyroid disorders. It is marked by lymphocytic infiltration and gradual destruction of thyroid tissue [1]. As a leading global cause of hypothyroidism, HT affects approximately 5%–10% of the general population and is significantly more common in women, with a female‐to‐male ratio of about 7:1 [2]. The condition typically manifests with nonspecific symptoms—such as fatigue, weight gain, and dry skin—that can notably reduce quality of life. Although common, the underlying mechanisms of HT are not fully elucidated, highlighting the importance of identifying novel biomarkers and associated risk factors.

In recent years, various hematological and biochemical markers have been investigated to evaluate the inflammatory state in HT. For example, red cell distribution width (RDW) is frequently increased in HT patients, indicating a possible link to chronic inflammation [3]. Vitamin *D* levels have also shown abnormal elevation in some studies, potentially reflecting dysregulation in immune function [4]. Moreover, multiple inflammation‐related ratios are often altered in individuals with HT, including the neutrophil‐to‐lymphocyte ratio (NLR), platelet‐to‐lymphocyte ratio (PLR), and uric acid to HDL cholesterol ratio (UHR) [5–7].

[5, 6]. These parameters may capture aspects of systemic inflammation, immune imbalance, and metabolic dysfunction, making them promising cost‐effective indicators for HT.

The albumin‐to‐globulin ratio (AGR) is a simple and widely available blood parameter that reflects the balance between albumin, which has anti‐inflammatory and antioxidant properties, and globulins, which include immunoglobulins involved in immune responses [7]. It serves as a marker of systemic inflammation and immune‐nutritional status and has been associated with various autoimmune diseases. For instance, in rheumatoid arthritis, AGR is reduced and correlates negatively with proinflammatory cytokines such as IL‐6 and TNF‐α. Nevertheless, evidence regarding AGR in HT is limited [8–13].

Tumor necrosis factor‐alpha (TNF‐α) is a key proinflammatory cytokine in HT, contributing to thyroid cell injury and immune cell recruitment [14]. Infiltrating immune cells further amplify inflammation, and TNF‐α can impair mitochondrial function, resulting in excessive reactive oxygen species (ROS) production and oxidative tissue damage [15]. As a major circulating antioxidant, albumin can inhibit TNF‐α–induced mitochondrial oxidative stress [16], thereby potentially attenuating cellular injury in HT. In parallel, the inflammatory microenvironment may activate the AKT/mTOR/NF‐κB pathway, suppress autophagy, and promote ROS accumulation and DNA damage. These changes facilitate Th17 differentiation and sustained cytokine release, forming a feed‐forward loop that accelerates thyroid destruction [15, 17]. Studies have indicated that lipopolysaccharide (LPS) can suppress albumin expression in hepatocytes via the NF‐κB pathway activation, while NF‐κB inhibitors (such as SN50) can restore albumin levels [18, 19]. A similar mechanism may exist in HT, potentially explaining the inverse relationship between NF‐κB activation and albumin levels, reflected by a decreased AGR [20]. With regard to apoptosis, thyroid follicular cells in HT undergo programmed cell death through the upregulation of the Fas/FasL system and downregulation of Bcl‐2 [14]. Similarly, nonylphenol (NP) has been shown to induce apoptosis in testicular cells via oxidative stress–mediated activation of the Fas/FasL–Bax/Bcl‐2 pathway [21]. Given that albumin can suppress oxidative stress, it may theoretically alleviate oxidative stress–induced apoptosis of thyroid cells in HT. However, this hypothesis requires further experimental validation.

Given the high prevalence of HT and the potential role of AGR in its pathogenesis, we conducted this study to investigate the association between AGR and HT. Utilizing data from the National Health and Nutrition Examination Survey (NHANES) 2007–2012, a nationally representative cross‐sectional survey, we aimed to provide robust evidence on this relationship. This study not only enhances our understanding of the role of AGR in HT but also explores its potential as a readily accessible marker for early detection and risk stratification in clinical practice.

## 2. Materials and Methods

### 2.1. Data Sources and Study Population

The NHANES, administered by the Centers for Disease Control and Prevention (CDC), is an ongoing, cross‐sectional survey aimed at evaluating the health and nutritional status of the civilian, noninstitutionalized US population. It integrates interviews, physical assessments, and laboratory testing, using a complex, multistage probability sampling strategy to achieve national representativeness. The study protocols are reviewed and approved by the Research Ethics Review Board of the National Center for Health Statistics (NCHS), and all participants provide informed consent prior to participation, ensuring ethical compliance and voluntary involvement. NHANES datasets are publicly accessible through the CDC website, and all personal identifiers are removed to preserve confidentiality. The anonymized nature of the data allows for secondary analysis, provided researchers follow the established usage and citation guidelines.

**FIGURE 1:**
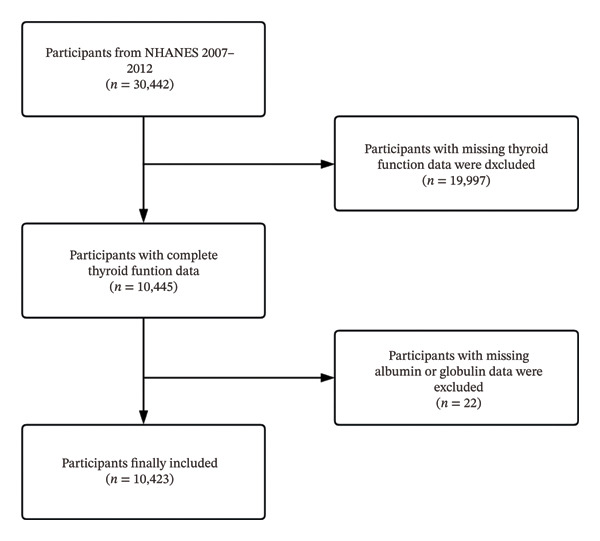
Flowchart of the study participants’ selection from the NHANES 2007–2012.

This study utilized data from the NHANES cycles conducted between 2007 and 2012, as thyroid function test results were only available during these survey years. The initial sample consisted of 30,442 participants from the NHANES 2007–2012 dataset. Participants without thyroid function data were excluded (*n* = 19,997), as these measurements were necessary for evaluating thyroid health. Additionally, participants missing albumin or globulin measurements required to calculate the AGR were excluded (*n* = 22). After applying these exclusion criteria, a total of 10,423 eligible participants were included in the final analysis (Figure 1). Moreover, this study was conducted in accordance with the revised 2013 Declaration of Helsinki.

### 2.2. Assessment of AGR and Its Components

The AGR, a key indicator of nutritional and physiological status, is derived by dividing serum albumin by globulin levels—both of which are critical components of serum proteins. Albumin concentration was assessed using the bichromatic digital endpoint technique on the DcX800 analyzer, where albumin reacts with bromocresol purple (BCP) to produce a measurable color change. Absorbance at 600 nm was recorded, with the resulting signal directly correlating with albumin concentration. This method is widely adopted in clinical evaluations of hepatic and renal function, as well as nutritional assessment, given albumin’s sensitivity to protein intake. Globulin levels were not measured directly; instead, they were calculated by subtracting albumin from total serum protein, which was quantified through the biuret method—a reaction between proteins and copper ions in alkaline solution, forming a color complex. Blood samples were obtained from NHANES participants aged 12 years and older under standardized conditions within Mobile Examination Centers (MECs). For participants scheduled in the morning, a minimum fasting period of 9 h was required prior to venipuncture to enhance the consistency and accuracy of metabolic and protein‐related measurements.

### 2.3. HT Diagnostic Methods

HT was identified through elevated thyroid‐specific autoantibodies, namely thyroglobulin antibodies (TgAb) and thyroid peroxidase antibodies (TPOAb). The diagnosis was established when TgAb concentrations were ≥ 4 IU/mL and/or TPOAb concentrations were ≥ 9 IU/mL. Quantification of both markers was performed using a sequential two‐step immunoenzymatic sandwich assay, involving incubation of the sample with paramagnetic particles coated with the corresponding protein—thyroglobulin for TgAb and thyroid peroxidase for TPOAb. After removing unbound materials, specific conjugates were added: a thyroglobulin–alkaline phosphatase conjugate for TgAb and a protein A–alkaline phosphatase conjugate for TPOAb. A chemiluminescent substrate (Lumi‐Phos 530) was then introduced, and the light generated was measured using a luminometer.

### 2.4. Covariates

We followed the methods of Lingli Chen et al. 2022 [22]. Based on previous literature and clinical importance, we identified a series of potential confounding variables due to their possible impact on the analysis. Demographic variables included age (years), sex (male/female), race/ethnicity (Mexican American, non‐Hispanic White, non‐Hispanic Black, or other), education level (≤ high school vs. > high school), and the poverty income ratio (PIR), classified as poor or not poor. Body mass index (BMI, kg/m^2^) was grouped into three categories: < 24.9, 24.9–30, and ≥ 30.

Clinical variables comprised the presence of hypertension and diabetes. Laboratory measurements included thyroid function indices and various biochemical markers, such as thyroid‐stimulating hormone (TSH, uIU/mL), free triiodothyronine (FT3, pg/mL), free thyroxine (FT4, ng/dL), TgAb (IU/mL), TPOAb (IU/mL), high‐density lipoprotein cholesterol (HDL‐C, mg/dL), low‐density lipoprotein cholesterol (LDL‐C, mg/dL), triglycerides (TG, mg/dL), total cholesterol (TC, mg/dL), alanine aminotransferase (ALT, U/L), aspartate aminotransferase (AST, U/L), alkaline phosphatase (U/L), fasting blood glucose (mg/dL), glycated hemoglobin (HbA1 c, %), albumin (g/dL), globulin (g/dL), and the AGR.

BMI was calculated by dividing body weight (kg) by the square of height (m^2^). Race/ethnicity and education were classified as stated above. Hypertension was defined as systolic blood pressure (SBP) ≥ 140 mmHg, diastolic blood pressure (DBP) ≥ 90 mmHg, or a self‐reported history of hypertension. Diabetes was identified based on self‐reported diagnosis, use of insulin or oral hypoglycemics, fasting glucose ≥ 126 mg/dL, or HbA1c ≥ 6.5%.

### 2.5. Statistical Analysis

All statistical analyses were performed using *R* software (Version 4.2) and EmpowerStats (Version 2.0), with a significance threshold set at *p* < 0.05. Continuous variables with a normal distribution were expressed as weighted means ± standard deviations (mean ± SD) and compared using independent *t*‐tests. For non‐normally distributed data, weighted medians and interquartile ranges (IQRs) were reported, and group differences were assessed using the Kruskal–Wallis rank‐sum test. Categorical variables were summarized as percentages and analyzed with chi‐square tests. Missing values were addressed through data imputation techniques.

Participants were stratified into quartiles based on their AGR to evaluate differences in baseline characteristics. To explore the association between AGR and the likelihood of HT, multivariable logistic regression was used to calculate odds ratios (ORs) and 95% confidence intervals (CIs). Three models were constructed for adjustment: Model 1: unadjusted; Model 2: adjusted for age, sex, and race/ethnicity; Model 3: further adjusted for potential confounders, including PIR, education level, hypertension, diabetes status, BMI, FT3, and FT4.

Interaction tests were conducted to assess effect modification by variables such as age, sex, race/ethnicity, marital status, education, hypertension, diabetes, BMI, and PIR, ensuring consistency of associations across subgroups. Additionally, smoothing curve fitting was employed—based on Model 3 adjustments—to assess potential nonlinear relationships between AGR and HT risk.

## 3. Results

### 3.1. Population Characteristics

The baseline characteristics of participants stratified by the presence or absence of HT are summarized in Table 1. Among the total population (*n* = 10,423), significant differences were observed between the HT group (*n* = 1355) and the non‐HT group (*n* = 9068) across several variables. Individuals with HT were older, with females being more prevalent in this group. Additionally, a higher proportion of HT patients were non‐Hispanic White. Marital status and education level also showed significant differences, with more HT participants being married or having education above high school.

**TABLE 1:** Baseline characteristics of participants by HT status (NHANES 2007–2012).

Characteristics	Total	Non‐Hashimoto’s thyroiditis	Hashimoto’s thyroiditis	*p* value
	(*n* = 10,423)	(*n* = 9068)	(*n* = 1355)	
Age, median (IQR) (years)	43.00 (36.00)	42.00 (37.00)	53.00 (31.00)	< 0.001
Age group (%)				< 0.001
< 40 years	4655 (44.66%)	4251 (46.87%)	404 (29.82%)	
40–60 years	2780 (26.67%)	2368 (26.11%)	412 (30.41%)	
≥ 60 years	2988 (28.67%)	2449 (27.00%)	539 (39.77%)	
Gender, *n* (%)				< 0.001
Male	5227 (50.15%)	4728 (52.36%)	479 (35.35%)	
Female	5196 (49.85%)	4320 (47.64%)	876 (64.65%)	
Race, *n* (%)				< 0.001
Mexican American	1805 (17.32%)	1566 (17.27%)	239 (16.64%)	
Non‐Hispanic White	4535 (43.50%)	3831 (42.25%)	704 (51.96%)	
Non‐Hispanic Black	2157 (20.70%)	2004 (22.10%)	153 (11.29%)	
Other	1926 (18.48%)	1667 (18.38%)	259 (19.11%)	
Marital status (%)				< 0.001
Married or living with a partner	5180 (49.70%)	4446 (49.03%)	592 (54.17%)	
Never married	1487 (14.27%)	1335 (14.72%)	117 (11.22%)	
Other	3756 (36.04%)	3287 (36.25%)	469 (34.61%)	
Education level, *n* (%)				< 0.001
High school or below	6303 (60.47%)	5553 (61.24%)	750 (55.35%)	
Above high school	4120 (39.53%)	3515 (38.76%)	605 (44.65%)	
BMI group, *n* (%)				0.034
< 24.9 kg/m^2^	3592 (34.46%)	3149 (34.73%)	443 (32.69%)	
24.9–30 kg/m^2^	3361 (32.25%)	2916 (32.16%)	445 (32.84%)	
≥ 30 kg/m^2^	3470 (33.29%)	3003 (33.11%)	467 (34.47%)	
PIR, *n* (%)				0.195
Poor	8863 (85.03%)	7727 (85.21%)	1136 (83.84%)	
Not poor	1560 (14.97%)	1341 (14.79%)	219 (16.16%)	
Hypertension, *n* (%)				< 0.001
Yes	7351 (70.53%)	6462 (71.26%)	889 (65.61%)	
No	3072 (29.47%)	2606 (28.74%)	466 (34.39%)	
Diabetes, *n* (%)				0.001
Yes	9342 (89.63%)	8162 (90.01%)	1180 (87.09%)	
No	1081 (10.37%)	906 (9.99%)	175 (12.91%)	
FT3, median (IQR) (pg/mL)	3.20 (0.57)	3.20 (0.51)	2.90 (0.50)	< 0.001
FT4, median (IQR) (ng/dL)	0.80 (0.20)	0.80 (0.20)	0.80 (0.20)	0.320
TSH, median (IQR) (uIU/mL)	1.53 (1.26)	1.49 (1.17)	1.97 (2.02)	< 0.001
aTG, median (IQR) (IU/mL)	0.60 (0.00)	0.60 (0.00)	4.20 (17.45)	< 0.001
aTPO, median (IQR) (IU/mL)	0.60 (1.20)	0.50 (0.80)	35.40 (161.05)	< 0.001
HDL‐C, median (IQR) (mg/dL)	50.00 (19.00)	50.00 (19.00)	51.00 (20.00)	< 0.001
LDL‐C, median (IQR) (mg/dL)	108.00 (47.00)	107.00 (47.00)	109.00 (46.00)	0.002
TG, median (IQR) (mg/dL)	16.2 (11.17)	10.25 (10.72)	5.31 (14.64)	0.008
TC, median (IQR) (mg/dL)	189.00 (42.40)	188.20 ± 42.32	194.78 ± 42.44	< 0.001
ALT, median (IQR) (U/L)	20.00 (11.00)	20.00 (11.00)	20.00 (11.00)	0.482
AST, median (IQR) (U/L)	23.00 (8.00)	23.00 (8.00)	23.00 (8.00)	0.375
Alkaline phosphatase, median (IQR) (U/L)	69.00 (30.00)	69.00 (31.00)	67.00 (27.00)	< 0.001
Glycohemoglobin (IQR) (%)	5.50 (0.60)	5.40 (0.60)	5.50 (0.70)	< 0.001
Albumin, median (IQR) (g/dL)	4.30 (0.40)	4.30 (0.40)	4.20 (0.40)	< 0.001
Globulin, median (IQR) (g/dL)	2.90 (0.60)	2.90 (0.60)	2.90 (0.50)	0.004
AGR, median (IQR)	1.48 (0.37)	1.48 (0.37)	1.46 (0.35)	< 0.001

*Note:* FT3, free triiodothyronine; FT4, free thyroxine; aTPO, anti–thyroid peroxidase antibody; TG, triglycerides; AST, aspartate aminotransferase; ALT, alanine aminotransferase.

Abbreviations: AGR, albumin‐to‐globulin ratio; aTG, anti–thyroglobulin antibody; BMI, body mass index; HDL‐C, high‐density lipoprotein cholesterol; LDL‐C, low‐density lipoprotein cholesterol; PIR, poverty‐to‐income ratio; TC, total cholesterol; TSH, thyroid‐stimulating hormone.

HT patients had elevated levels of HDL‐C, LDL‐C, TC, and TG, as well as higher TSH levels. Conversely, FT3 levels were lower in the HT group compared to non‐HT participants. Significant differences were also observed in BMI distribution, with a greater percentage of HT patients classified as overweight or obese (BMI ≥ 30 kg/m^2^). Furthermore, HT patients had a lower prevalence of hypertension and diabetes compared to their non‐HT counterparts.

### 3.2. Association Between AGR and HT

To assess the association between the AGR and the risk of HT, a multivariable logistic regression analysis was performed, as shown in Table 2. In the unadjusted model (Model 1), each one‐unit increase in AGR was associated with a 37% reduction in HT risk (OR = 0.63; 95% CI: 0.52–0.78; *p* < 0.05). After adjusting for age, sex, and race/ethnicity (Model 2), the association remained significant, indicating a 27% lower risk (OR = 0.73; 95% CI: 0.59–0.92; *p* < 0.05). In the fully adjusted model (Model 3), which additionally accounted for socioeconomic factors, comorbidities, and thyroid function markers, AGR was still inversely associated with HT, with a 29% risk reduction per unit increase (OR = 0.79; 95% CI: 0.63–0.99; *p* < 0.05). These consistent findings across all models highlight a robust negative relationship between AGR and the likelihood of developing HT.

**TABLE 2:** The association between AGR and HT.

Exposure	Model 1 OR (95% CI)	*p* value	Model 2 OR (95% CI)	*p* value	Model 3 OR (95% CI)	*p* value
AGR	0.63 (0.52, 0.78)		0.73 (0.59, 0.92)		0.79 (0.63, 0.99)	
Q1 (0.39–1.30)	Reference		Reference		Reference	
Q2 (1.31–1.48)	0.88 (0.75, 1.03)	0.1122	0.90 (0.76,1.06)	0.1867	0.90 (0.76, 1.06)	0.1983
Q3 (1.49–1.66)	0.89 (0.76, 1.04)	0.1530	0.94 (0.80, 1.13)	0.4946	0.97 (0.83, 1.17)	0.8653
Q4 (1.67–3.61)	0.68 (0.58, 0.80)	< 0.0001	0.77 (0.65, 0.92)	0.0034	0.80 (0.67, 0.96)	0.0164
*p* for trend	< 0.0001		0.0079		0.0425	

### 3.3. Sensitivity Analysis

**FIGURE 2:**
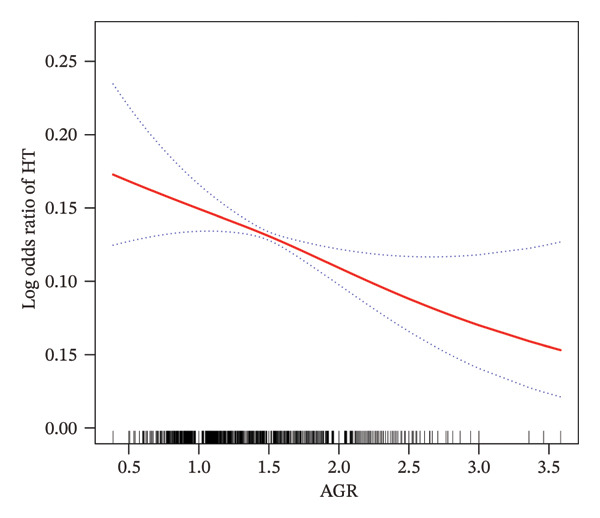
The association between AGR and HT. The red solid line represents the smooth curve fitting between the variables, while the blue dashed lines indicate the 95% confidence interval of the fitted results.

To assess the robustness of our findings and explore potential nonlinear effects, AGR was categorized into quartiles—a standard approach in epidemiological studies when clinical cutoffs are undefined [23]. This method also aligns with the distribution of AGR in our population (median = 1.48, IQR = 0.37), ensuring balanced subgroup comparisons. As part of the sensitivity analysis, the AGR was categorized into quartiles to assess the robustness and reliability of the findings. The association patterns remained consistent with those observed when AGR was analyzed as a continuous variable, as confirmed by a significant *p* value for trend (Table 2). To further evaluate the potential linear association between AGR and HT risk, a smoothed curve was generated based on the fully adjusted model (Model 3). As shown in Figure 2, the fitted curve indicates a clear inverse relationship between AGR and the likelihood of developing HT.

### 3.4. Subgroup Analysis

**FIGURE 3:**
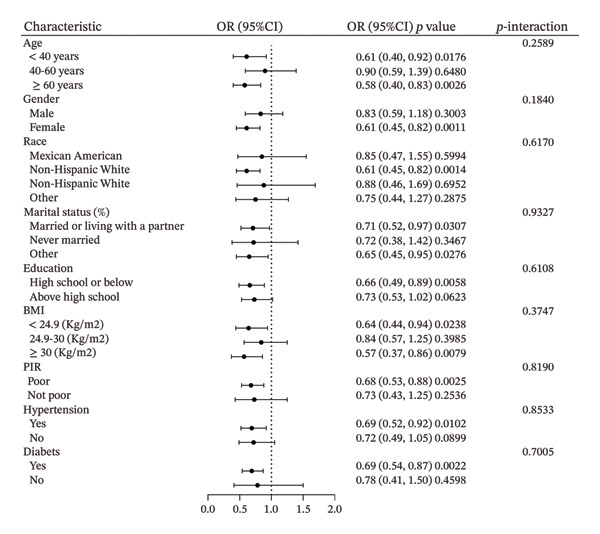
Stratified associations between AGR and HT. Adjusted for age, gender, race, marital status, education level, BMI, PIR, hypertension, diabetes.

To further explore the association between AGR and the risk of HT, subgroup and interaction analyses were conducted based on stratification by age, sex, race/ethnicity, marital status, educational background, hypertension, diabetes, BMI, and PIR. The stratified results showed no significant effect modification in any subgroup. Specifically, interaction *p* values were nonsignificant for age (< 40, 40–60, ≥ 60 years; *p* = 0.2589), sex (male vs. female; *p* = 0.1840), race (Mexican American, non‐Hispanic White, non‐Hispanic Black, other; *p* = 0.6170), marital status (living with partner, never married, other; *p* = 0.9327), education level (high school or less vs. above high school; *p* = 0.6108), BMI (< 24.9, 24.9–30, ≥ 30 kg/m^2^; *p* = 0.3747), PIR (poor vs. not poor; *p* = 0.8190), presence of hypertension (*p* = 0.8533), and diabetes (*p* = 0.7005). As presented in Figure 3, these findings indicate that the inverse association between AGR and HT is consistent across all evaluated subgroups.

### 3.5. Association Between AGR and TPOAb, TgAb

Given the critical role of thyroid autoantibodies in the pathogenesis and progression of HT, we investigated the associations between AGR levels and two key thyroid autoantibodies—TPOAb and TgAb—to evaluate whether AGR could serve as a potential marker of autoimmune activity. Multivariable regression analysis based on Model 3 (Table 3) revealed a significant linear negative association between AGR and TPOAb levels (*β* = −8.95; 95% CI: −15.07 to −2.83; *p* = 0.002). However, no statistically significant association was observed between AGR and TgAb levels (*β* = −2.96; 95% CI: −9.28 to 3.37; *p* = 0.4583).

**TABLE 3:** The association between AGR and TPOAb, TgAb.

Exposure	Model 1 OR (95% CI)	*p* value	Model 2 OR (95% CI)	*p* value	Model 3 OR(95% CI)	*p* value
aTPO						
AGR	−15.19 (−20.98, −9.41)		−11.05 (‐17.35,‐4.75)		−8.95 (−15.07, −2.83)	
Q1 (0.39–1.30)	Reference		Reference		Reference	
Q2 (1.31–1.48)	−6.52 (−11.33, −1.71)	0.0079	−5.74 (−10.58, −0.90)	0.0200	−6.05 (−10.74, −1.35)	0.0178
Q3 (1.49–1.66)	−6.72 (−11.50, −1.93)	0.0059	−5.07 (−9.99, −0.16)	0.0433	−4.15 (−8.92, −0.63)	0.0436
Q4 (1.67–3.61)	−13.26 (‐17.95, −8.57)	< 0.0001	−9.93 (−14.97, −4.87)	0.0001	−8.58 (−13.48, −3.69)	0.0004
*p* for tend	< 0.0001		0.0003		0.0020	

aTG						
AGR	−5.93 (−11.77, −0.09)		−4.00 (−10.38, 2.36)		−2.96 (−9.28, 3.37)	
Q1 (0.39–1.30)	Reference		Reference		Reference	
Q2 (1.31–1.48)	−3.50 (−8.82, 1.35)	0.1568	−2.91 (−7.80, 1.97)	0.2427	−3.06 (−7.91, 1.79)	0.2163
Q3 (1.49–1.66)	−4.00 (−9.30, 0.16)	0.1043	−2.96 (−7.93, 2.01)	0.2425	−2.49 (−7.43, 2.44)	0.3017
Q4 (1.67–3.61)	−4.57 (−14.47, 0.44)	0.0587	−2.88 (−7.98, 2.21)	0.2670	−2.24 (−7.30, 2.82)	0.3847
*p* for tend	0.0653		0.3004		0.4583	

To verify the stability of the results, a sensitivity analysis was conducted by dividing AGR into quartiles. Individuals in the highest quartile (Q4) had significantly lower TPOAb levels than those in the lowest quartile (Q1) across all adjustment models. In contrast, no consistent association was found between AGR quartiles and TgAb levels.

In addition, smooth curve fitting was applied to explore potential nonlinear relationships between AGR and the two thyroid autoantibodies (Figure 4). The results demonstrated a linear negative association between AGR and TPOAb levels, consistent with the regression analysis. Conversely, although a downward trend was observed between AGR and TgAb levels, this relationship did not reach statistical significance.

FIGURE 4Smooth curve fitting analysis of the association between AGR and thyroid autoantibodies. (a) The relationship between AGR and TgAb levels; (b) the relationship between AGR and TPOAb levels.

**Figure:**
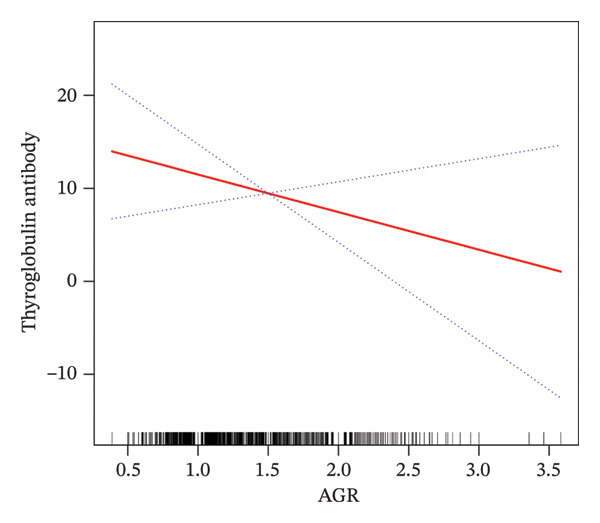
(a)

**Figure:**
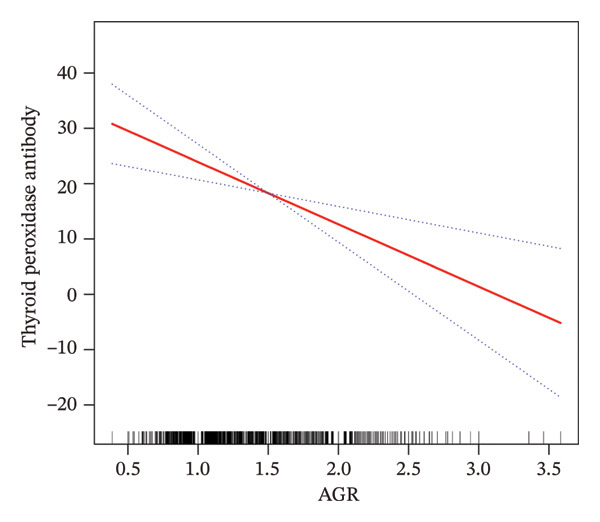
(b)

## 4. Discussion

This study, based on NHANES data, found a significant linear inverse association between the AGR and the risk of HT. After multivariable adjustment for potential confounders, the association remained robust: Lower AGR levels were associated with a higher likelihood of HT, and each one‐unit increase in AGR corresponded to an approximately 30% reduction in HT risk, suggesting the potential clinical relevance of AGR. Given that AGR is a routine, readily available, and low‐cost laboratory parameter and may partially reflect nutritional status and systemic inflammation, it could be useful for HT risk assessment and early identification. Further analyses showed a significant inverse correlation between AGR and TPOAb levels, whereas the association with TgAb did not reach statistical significance; curve fitting, sensitivity, and subgroup analyses yielded consistent results, supporting the stability of our findings. Leveraging the nationally representative NHANES sample and assessing both major thyroid autoantibodies, this study enhances statistical power and improves the generalizability of the findings to the US population.

Previous studies have examined nutritional and metabolic factors in the development of HT. Using NHANES data, one study reported an inverse association between vitamin C intake and HT‐related hypothyroidism [24], supporting a potential link between antioxidant status and autoimmune inflammation [25–27]. Another study found that higher serum iron levels were associated with a lower risk of HT in women of reproductive age [28], and iron was inversely related to TPOAb, while its association with TgAb appeared nonlinear. In contrast to these studies focusing on individual nutrients or trace elements, our study highlights the AGR—a routinely available composite index reflecting both nutritional status and systemic immune activity—and demonstrates its significant association with HT risk. Together with prior evidence linking lower AGR to inflammatory‐related conditions [29], our findings extend the current literature and suggest that AGR may provide an additional, clinically accessible window into the metabolic–immune milieu underlying HT.

Albumin is the most abundant plasma protein synthesized in the liver and contributes to oncotic pressure maintenance, transport functions, and anti‐inflammatory activity [23, 30]. In systemic inflammatory conditions, cytokines such as IL‐6 and TNF‐α may suppress hepatic albumin synthesis and increase vascular permeability, which can lower circulating albumin concentrations [31]. Therefore, lower albumin may reflect an inflammatory or suboptimal nutritional milieu that is biologically compatible with heightened autoimmune activity [32–34]. Globulins comprise *α*1‐, *α*2‐, *β*‐, and *γ*‐globulin fractions, with *γ*‐globulins (immunoglobulins) representing adaptive immune activation [35–37]. In HT, autoantigen‐driven B‐cell responses are accompanied by the production of thyroid autoantibodies (TPOAb and TgAb), and immunoglobulin‐rich globulin fractions may increase accordingly [38, 39]. Immune complex formation and complement activation have been implicated in thyroid tissue injury in autoimmune thyroiditis [27, 40, 41]. Thus, elevated globulin levels are not only a pathological feature of HT but may also accelerate disease progression by amplifying inflammation and immune responses [42, 43]. As a composite index, AGR captures the balance between albumin and globulin and may serve as a nonspecific marker of systemic immune activity [44]. In our analyses, lower AGR was associated with higher odds of HT and was inversely correlated with TPOAb, suggesting that AGR may track antibody‐related immune activity in this population. The absence of a statistically significant association with TgAb may reflect heterogeneity in TgAb‐related pathways or limited power for that endpoint. Collectively, these associational findings underscore the hypothesis‐generating nature of our study, highlighting potential links for future mechanistic investigation rather than elucidating direct causal pathways.

Several limitations should be acknowledged. As this study employed a cross‐sectional design, it is not possible to determine whether low AGR levels are a causative factor for HT, or whether the presence of HT induces inflammation that subsequently affects AGR levels, indicating a potential issue of reverse causality. Therefore, further longitudinal or prospective studies are necessary to confirm the predictive value of AGR in the development of HT. In addition, this study defined HT based on antibody positivity, and consequently did not identify seronegative HT (SN‐HT) patients. Previous research has indicated that SN‐HT may account for approximately 20% of all HT cases [45]. This subgroup may exhibit different characteristics in terms of AGR levels or inflammatory responses, suggesting that the current findings may not be fully generalizable to this population. Finally, although the NHANES data are nationally representative within the United States, the generalizability of these findings to populations in other countries or to specific subgroups with distinct cultural or regional characteristics may be limited. Future research should include prospective cohort studies to validate the association between AGR and HT incidence, longitudinal studies to investigate whether declining AGR levels predict the onset of HT, and interventional studies to explore whether modifying AGR levels can influence HT risk or disease progression.

Lower AGR levels were independently associated with higher odds of HT, and AGR showed a significant inverse correlation with TPOAb. Given its accessibility and low cost, AGR may be a pragmatic biomarker for identifying individuals at increased risk of HT and for reflecting antibody‐related immune activity. Notably, multivariable adjustment with flexible curve fitting and sensitivity analyses helped characterize the pattern and robustness of the AGR–HT association, providing a novel epidemiologic perspective that can inform hypothesis generation regarding potential immuno‐inflammatory pathways. However, because this study is observational and cross‐sectional and does not assess direct cellular or molecular processes, mechanistic and prospective studies are warranted to determine whether changes in AGR precede HT onset and to clarify underlying biological mechanisms.

## Author Contributions

All authors have made substantial contributions to this study. Wenjun Wu and Jing Jin contributed equally to this work, including study design, data analysis, and manuscript drafting. Nana Qin and Dutian Xu were involved in data collection, statistical analysis, and literature review. Fangxu Liu contributed theoretically to the hypothesis formation, assisted in restructuring the logic of the mechanistic discussion, and provided key references during the revision process. Yuanchen He and Baoyin Li supervised the study, provided critical revisions, and were responsible for correspondence.

## Funding

The authors did not receive support from any organization for the submitted work.

## Disclosure

All authors have read and approved the final manuscript and agree to be accountable for all aspects of the work.

## Ethics Statement

All NHANES protocols received approval from the ethical review committee of the National Center for Health Statistics.

## Conflicts of Interest

The authors declare conflicts of interest.

## Data Availability

The datasets presented in this study can be found in online repositories.
